# Defining ‘usual care' in trials of complex interventions

**DOI:** 10.1186/1745-6215-14-S1-P7

**Published:** 2013-11-29

**Authors:** Julia Sanders, Marie-Jet Bekkers, Rebecca Cannings-John, Sue Channon, Mike Robling

**Affiliations:** 1 South East Wales Trials Unit, Cardiff, UK

## Background

Defining ‘usual care' in studies of health services is challenging as it comprises a large variety of locally variable services. Building Blocks is trialling the Family Nurse Partnership Programme (FNP) in England. FNP is a complex intervention provided to first-time teenage mothers in the community setting. It was developed and previously trialled in the USA where support services are very different. To fully interpret trial results, particularly when making international comparisons, insight into the control condition is essential.

## Methods

We used a mixed methods approach to 'map' services provided to teenage mothers in our 18 trial sites. Initially, a variety of local professional stakeholders described characteristics of health and social services across five domains. This data informed the construction of a follow-up web-based survey filled in by a single local informant per site. Finally, in-depth telephone interviews with a sample of these informants sought to highlight changes in service provision over the course of the trial.

## Results

The outcomes of the initial mapping exercise lacked consistency but were useful to inform both the construction of the follow-up survey and the main trial data collection questionnaires. The web-survey results provided snapshots of service provision per site and the interviews contextualised local changes.

## Discussion

Variation of usual care needs to be acknowledged in the interpretation of results, particularly in the assessment of the study's generalisability. Our service mapping exercise provided global insight into within-site variations and changes over time. Practical limitations prevented exploring details of uptake and local criteria for entitlement.

